# Oncoplastic Surgery for Upper/Upper Inner Quadrant Breast Cancer

**DOI:** 10.1371/journal.pone.0168434

**Published:** 2016-12-28

**Authors:** Joseph Lin, Dar-Ren Chen, Yu-Fen Wang, Hung-Wen Lai

**Affiliations:** 1 Comprehensive Breast Cancer Center, Department of Surgery, Changhua Christian Hospital, Changhua, Taiwan; 2 Cancer Research Center, Changhua Christian Hospital, Changhua, Taiwan; 3 School of Medicine, Chung Shan Medical University, Taichung, Taiwan; 4 Endoscopic & Oncoplastic Breast Surgery Center, Changhua Christian Hospital, Changhua, Taiwan; 5 School of Medicine, National Yang Ming University, Taipei, Taiwan; Fu Jen Catholic University, TAIWAN

## Abstract

Tumors located in the upper/upper inner quadrant of the breast warrant more attention. A small lesion relative to the size of breast in this location may be resolved by performing a level I oncoplastic technique. However, a wide excision may significantly reduce the overall quality of the breast shape by distorting the visible breast line. From June 2012 to April 2015, 36 patients with breast cancer located in the upper/upper inner quadrant underwent breast-conservation surgery with matrix rotation mammoplasty. According to the size and location of the tumor relative to the nipple-areola complex, 11 patients underwent matrix rotation with periareolar de-epithelialization (donut group) and the other 25 underwent matrix rotation only (non-donut group). The cosmetic results were self-assessed by questionnaires. The average weights of the excised breast lumps in the donut and non-donut groups were 104.1 and 84.5 g, respectively. During the 3-year follow-up period, local recurrence was observed in one case and was managed with nipple-sparing mastectomy followed by breast reconstruction with prosthetic implants. In total, 31 patients (88.6%) ranked their postoperative result as either acceptable or satisfactory. The treated breasts were also self-evaluated by 27 patients (77.1%) to be nearly identical to or just slightly different from the untreated side. Matrix rotation is an easy breast-preserving technique for treating breast cancer located in the upper/upper inner quadrant of the breast that requires a relatively wide excision. With this technique, a larger breast tumor could be removed without compromising the breast appearance.

## Introduction

**Fig 1:**
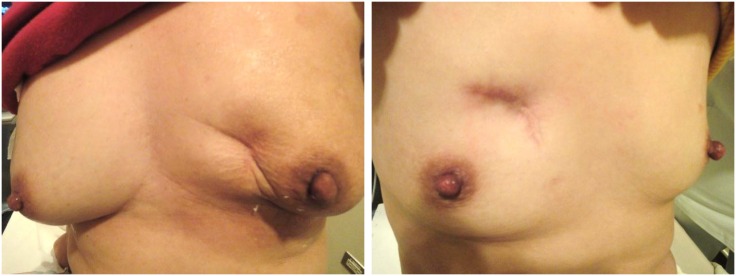
Two representative cases of breast deformity after extensive excisions in the upper inner quadrant.

The approval of breast-conserving surgery (BCS) by the World Health Organization Committee of Investigations for Evaluation of Methods of Diagnosis and Treatment of Breast Cancer since 1996 [1] has offered an alternative treatment method for early-stage breast cancer besides radical mastectomy. In several randomized studies by Veronesi et al [2–4], it has been demonstrated that BCS gave an overall survival rate equivalent to that by mastectomy. Moreover, BCS offered higher quality of life by reducing the impact of psychosocial adjustment, body image, and sexual function caused by mastectomy [5]. The success of BCS is based upon the foundation of removing the tumor with adequate margins together with post-operative radiotherapy. BCS delivers good clinical outcomes and has become the preferred treatment for early-stage breast cancer. The ultimate goals of BCS for breast cancer are to completely resect the breast tumor with adequate margins and to simultaneously preserve the natural shape of the breast. As contradictory as it sounds, it can be difficult to remove a tumor which is large relative to the size of the breast without sacrificing esthetics. Deformity can often occur in medium- to large-sized breasts (Fig 1) without the proper surgical technique, and this can prompt a recommendation of mastectomy to the patient. Furthermore, in conventional BCS, approximately 5%–18% of cases had positive margins, which led to high re-excision rates [6, 7]. These high re-excision rates can be significant in terms of complications, morbidity, and deformity.

The term “oncoplastic” was first introduced in the literature by Gabka et al [8] in 1997 to expand the spectrum of the indication for BCS. Oncoplastic surgery (OPS) involves more than just the combination of oncologic principles with plastic techniques [6]. Using various mammoplastic methods of remodeling the remaining breast tissue, surgeons can perform larger excisions with free margins, thereby reducing the rate of re-excision [9]. As a general rule, 80 g of breast tissue is the maximum weight that can be removed from a medium-sized breast without resulting in deformity. Although the average specimen from BCS weighs 20–40 g, all OPS studies have demonstrated that an average of 200 g up to 1 000 g or more can be removed from a medium to large-sized breast during BCS with no cosmetic compromises [10]. In 2010, Clough et al [11] developed an Atlas and OPS guideline to assist surgeons in choosing the optimal approach for each individual patient. Among others, lesions located in the upper/upper inner quadrant of the breast deserve the most attention; a wide excision in this location can have a significant impact on the overall quality of the breast shape by distorting the visible breast line. Thus far, there has not been a standard level II oncoplastic procedure developed by Clough et al. to reliably address this difficult-to-treat area. Here we report on the use of “matrix rotation” to improve cosmetic outcome in patients with breast tumors located in the upper/upper inner quadrant.

## Materials and Methods

### Patients

This is a retrospective review of 36 Taiwanese female patients who underwent BCS followed by immediate reconstruction employing matrix rotation mammoplasty at the Changhua Christian Hospital from June 2012 to April 2015. All surgical procedures were performed by one senior breast surgeon. The enrollment criteria for this study were as follows: (1) breast cancer patients who were candidates for BCS, (2) tumor size of no more than 5 cm in transverse diameter in a small-to-moderate-sized breast, (3) the tumors were located in the upper/upper inner quadrant, and (4) a fatty breast unsuitable for extensive dissection using level I OPS. The Changhua Christian Hospital Institutional Review Board approved the retrospective study and the written informed consent was waived (CCH IRB No. 160110). Therefore, this study required neither patient approval nor informed consent for the review of medical records. The patients’ consents were obtained for photographing and publishing purposes. Any information regarding the personal identity of the patient in each photograph was removed before publishing. Furthermore, the patients’ medical records and personal information were anonymized and de-identified prior to analysis.

**Fig 2:**
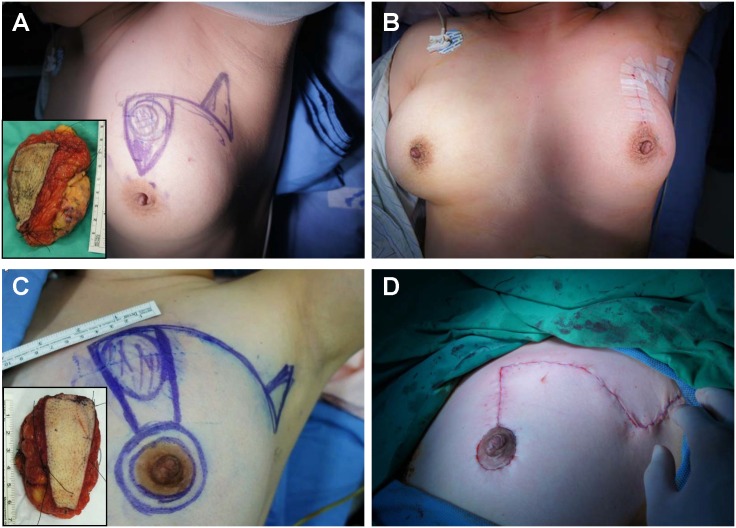
Patients were divided into either the non-donut or donut group, depending on the distance between the tumor and NAC. (A, B) A representative case of the non-donut group. The tumor was located 3 cm away from NAC. The patient underwent matrix rotation without de-epithelialization. The specimen measured 10 × 7.5 cm and weighed 126 g. (C, D) Patient with a tumor located at the 10 o’clock position underwent matrix rotation with de-epithelialization. Preoperative drawing with postoperative scar. The specimen measured 7 × 4.5 cm and weighed 56 g.

The 36 patients were assigned to either the non-donut or donut group, depending on the size and location of the tumor relative to the nipple-areola complex (NAC). The non-donut method was employed for those tumors which could be resected by a reverse-triangular shaped *en bloc* resection with negative margins (Fig 2A and 2B). For tumors located less than 3 cm away from NAC or if safety margins could not be obtained by non-donut *en bloc* resection, the donut method was used, which involved periareolar de-epithelialization and *en bloc* resection in a rectangular shape (Fig 2C and 2D). Eleven patients were assigned to the donut group and 25 to the non-donut group.

### Oncoplastic techniques

**Fig 3:**
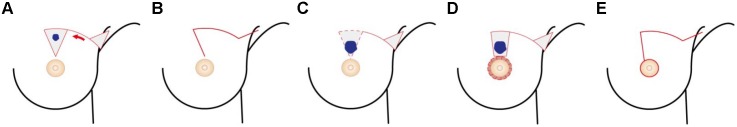
Surgical technique of the matrix rotation. The non-donut method was employed for those tumors which could be resected by a reverse-triangular shaped *en bloc* resection with negative margins (A). A concave incision was made along the lateral border of the breast from the tumor site to the midaxillary line, followed by a small triangular-shaped incision in the axilla to facilitate lymph node management and subsequently flap advancement (B). For tumors located less than 3 cm away from NAC or if safety margins could not be obtained by non-donut *en bloc* resection (C), the donut method was used which involved periareolar de-epithelialization and *en bloc* resection in a rectangular shape (D and E).

Under general anesthesia, the patients were placed in the supine position with the arm on the operative side abducted. All operative sites had been marked before the operation. For the non-donut group, an inverse triangular shaped *en bloc* resection that included the tumor with at least 1 cm of safe margin was performed. It was followed by the extension of a concave incision from the tumor site along the lateral border of the breast to the midaxillary line. A small triangular-shaped resection was then made in the axilla to facilitate lymph node management and later rotation flap advancement (Fig 3A and 3B). For tumors located less than 3 cm away from NAC or if safety margins could not be obtained by non-donut *en bloc* resection (Fig 3C), the donut method was employed. For the donut group, the tumor was removed by periareolar de-epithelialization and a rectangular shaped *en bloc* resection, followed by the same procedures in the axilla as those performed in the non-donut method (Fig 3D and 3E). No postoperative drainage placement in the breast or axilla was necessary, except in those patients who underwent axillary lymph node dissection. The details of matrix rotation are illustrated in Fig 3.

### Questionnaire

Thirty-five of the 36 patients who underwent matrix rotation were asked to fill out a questionnaire adapted from that used by Chan et al [12] for the subjective assessment of satisfaction with the outcome (one patient was lost to follow-up). The questionnaires were sent out in August 2015, after patients have completed their radiation therapies. They included the following questions: (1) regarding the patients’ satisfaction with the postoperative appearance, (2) how the treated breast compared to the other breast from the patients’ viewpoint, (3) if they would have chosen another kind of breast surgery, and (4) if they would consider any further surgical procedures to reshape the treated breast.

## Results

The mean age of the 36 patients studied was 54.6 years (range, 25–89 years). The mean length of hospital stay was 3.2 days (range, 2–7 days). Patients’ pathologic stages were as follows: ductal carcinoma in situ (n = 3), IA (n = 10), IB (n = 1), IIA (n = 14), IIB (n = 5), IIIA (n = 2), and IIIB (n = 1) (Table 1).

**Table 1:** Characteristics of the patients who underwent matrix rotation procedures.

	Donut (n = 11)	Non-Donut (n = 25)
**Age (year)**		
*Mean (range)*	50.0 (35–65)	53.9 (25–89)
*Median*	50	52
**Mean length of hospital stay (days)**	3.7	2.9
**Mean resected tissue weight (range, g)**	104.1 (54–272)	84.5 (25–263)
**Tumor stage**		
*In situ*	1	2
*IA*	2	8
*IB*	1	0
*IIA*	2	12
*IIB*	3	2
*IIIA*	2	0
*IIIB*	0	1
**Complications**		
*Hematoma*	0	0
*Seroma*	0	0

The mean resected tissue weight for the 11 patients in the donut group was 104.1 g (range, 54–272 g); size ranged from 6.0 × 5.5 cm to 12 × 10 cm. In the non-donut group, the mean resected tissue was 84.5 g (range, 25–263 g); size ranged from 5.5 × 4.5 cm to 11.5 × 9.5 cm.

The time taken for the operation was approximately 50 min on an average, which included breast-conserving surgery with sentinel lymph node biopsy and surgical suture. Blood loss in general was minimal (approximately 30 mL). There was only one patient with close margins (less than 1 mm) who subsequently underwent re-excision 1 week after the initial operation, and her final pathology report showed no residual cancer. During the 3-year follow-up period, no wound complications, such as hematoma or seroma formation, were observed. However, local recurrence was observed in one case, and it was managed with nipple-sparing mastectomy followed by breast reconstruction with prosthetic implants.

Questionnaires were sent to 35 patients after they completed their radiation therapy; one patient was lost to follow-up. Cosmetic outcomes were self-reported to be excellent in 3 cases (8.6%), satisfactory in 14 cases (40.0%), acceptable in 17 cases (48.6%), and poor in 1 case (2.9%). Overall, a total of 31 patients (88.6%) ranked their postoperative result as either acceptable or satisfactory. The treated breasts were also self-evaluated by 27 patients (77.1%) to be nearly identical to or just slightly different from the untreated side. A summary of the questions and the results is shown in Table 2.

**Table 2:** Patient questionnaire results after oncoplastic breast-conserving surgery (n = 35; 1 patient was lost to follow-up).

Patient questionnaire	Donut (n = 11)		Non-Donut (n = 24)	
	N	%	N	%
**Are you satisfied with your postoperative appearance?**				
*Dissatisfied*	0	0	1	4.2
*Acceptable*	6	54.6	11	45.8
*Satisfied*	4	36.4	10	41.7
*Very satisfied*	1	9.1	2	8.3
**Compared with the untreated breast, how different is the treated breast?**				
*Seriously distorted*	2	18.2	0	0
*Clearly different from the untreated breast*, *but not seriously distorted*	3	27.3	3	12.5
*Slightly different from untreated breast*	4	36.4	9	37.5
*Nearly identical*	2	18.2	12	50.0
**If you can choose again, will you have another kind of breast surgery? (e.g., mastectomy with breast reconstruction)**				
*Yes*	0	0	3	12.5
*Uncertain*	0	0	2	8.3
*No*	11	100.0	19	79.2
**Will you consider further surgery to reshape the treated breast?**				
*Yes*	2	18.2	0	0
*Uncertain*	0	0	2	8.3
*No*	9	81.8	22	91.7

## Discussion

Oncoplastic breast surgery is safe in terms of local recurrence and survival rates and is comparable to conventional breast-conserving management of tumors in difficult-to-treat locations and high in volume [13, 14]. Its application is influenced by several factors, such as tumor size, tumor location, breast size, resection volume, and radiation therapy [15, 16]. Several techniques have been developed for each breast quadrant; however, the upper inner quadrant is still a less favorable location. Grisotti et al defines the upper inner quadrant as the “no man’s land” as full-thickness excision with the skin of the quadrant can cause upward displacement of NAC [17]. For moderate resections at this location, OPS level I involving simple reshaping techniques can be safely utilized unless the treated breast is categorized as BIRADS ½ because dual-plane undermining can sometimes lead to complication such as fat necrosis or glandular necrosis. Anderson et al have utilized batwing mastopexy to address tumors in the upper inner quadrant, which involves two closely similar half-circle incisions with angled wings on each side of the areola [18]. His approach is reproducible; however, the procedure will not only cause some lifting of the nipple, which can lead to asymmetry, but can also lead to nipple necrosis if the dissection extends up to a higher position behind the nipple. The modified round block mammoplasty introduced by Chen in 2014 [19] also gave excellent results for lesions in the upper quadrant. It is a good surgical choice for all quadrant-located breast cancers, particularly in small-to-medium-sized breasts with mild-to-moderate ptosis. The crescent mastopexy resection involves the excision of a crescent-shaped area of skin and glandular tissue from the superior border of the areola and removal of the cancerous lesion in the central breast superior to NAC. This technique allows the removal of skin overlying a tumor in the superficial breast, ensuring a clear superficial margin. However, crescent mastopexy is only ideal for lesions located in between the 10 and 1 o’clock the periareolar position. Any lesions that are more medial or lateral would cause deviation of NAC [20]. The inferior pedicle mammoplasty via an inverted-T incision can be also be used for tumors located within the superior aspect of the breast. The tumor is removed *en bloc* with an inverted-T incision followed by elevation of the de-epithelialized inferior pedicle and re-approximation of the medial and lateral glandular flaps [21].

**Fig 4:**
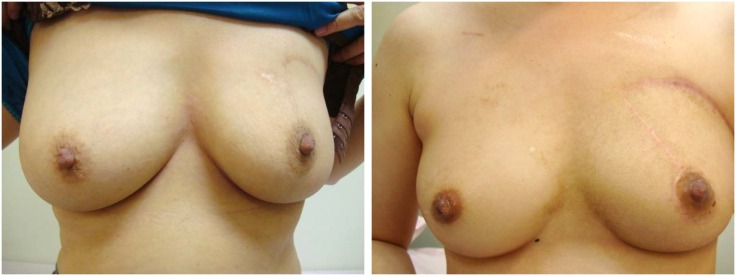
One year after the operation. The long scars faded away.

Although several innovative techniques, such as crescent, batwing, hemi-batwing excisions, and modified round-block mammoplasty, were reported to be effective with good esthetic results [19, 22], there has not been a standard level II oncoplastic procedure developed by Clough et al to address this difficult-to-treat area. Our technique is divided into a two-step operation. First, a wedge-shaped block of tissue containing the tumor with or without a donut of skin around the nipple is removed and second, reconstruction with matrix rotation flap advancement is performed. “Matrix rotation” can be another potential choice of technique for upper inner lesions because of the following advantages: (1) full-thickness excision and removal of overlying skin can be easily performed, even by a general surgeon, (2) the operation can be performed in less than 50 min, (3) manipulation of the contralateral nipple is not necessary, (4) axillary dissection can be performed easily, (5) multiple teams working closely during the entire process are not required, (6) blood loss is minimal (approximately 30 mL on average), and (7) wound complications such as hematoma or seroma formation are not observed. Therefore, matrix rotation may be a good alternative method if the operation requires more extensive resections or if the breast is mostly composed of fatty tissue (BIRADS ½). The only drawback of this procedure is a relatively long S scar. However, most of those scars will fade after some years (Fig 4).

It is estimated that approximately 5%–25% of patients could have a poor cosmetic outcome after OPS [23, 24]. In 1999, Al-Ghazal and Blamey illustrated data demonstrating no correlation between scar length and satisfaction [25]. Although other studies have reported a significant influence of scar length on overall patient satisfaction, these studies do not all refer to OPS. We also focused on esthetics, particularly from the patient’s viewpoint, to gather more information for a better understanding of patient perceptionand comfort after the surgical procedure. Fourteen patients were satisfied with their cosmetic results, and only three would choose other type of breast surgeries, such as mastectomy followed by total reconstruction, if they could choose again. Furthermore, only two patients considered having further surgeries to reshape their treated breasts for esthetic means. Younger age was significantly associated with either choosing another surgical method or further reshaping the treated breast. This could be explained as younger patients have higher expectations than older patients, and therefore, they tend to rank the cosmetic result lower in cases of smaller deviations. Overall, majority of our patients appeared to be either satisfied or in acceptance of the treated breast despite the S-shaped incision and scar.

Therefore, matrix rotation is an easy, safe, and reliable technique for treating breast cancer located in the upper/upper inner quadrant that requires a relatively wide excision. Using this technique, a larger breast tumor could be removed without interfering with the overall breast appearance. This matrix rotation technique may become more widely available and perhaps be accepted as a standard procedure for upper quadrant located breast tumors, particularly in small-to-medium-sized breasts.
